# First metatarsal single-screw minimally invasive chevron-akin osteotomy: A cost effective and clinically reliable technique

**DOI:** 10.3389/fsurg.2022.1047168

**Published:** 2023-01-05

**Authors:** Xueqian Li, Jieyuan Zhang, Shaoling Fu, Cheng Wang, Fan Yang, Zhongmin Shi

**Affiliations:** Department of Orthopedic Surgery, Shanghai Sixth People's Hospital, Shanghai Jiao Tong University, Shanghai, China

**Keywords:** hallux valgus, minimally invasive osteotomy, chevron, AKIN, cost-effectiveness

## Abstract

**Purpose:**

The common disease hallux valgus results in foot discomfort and dysfunction. Less soft tissue damage and faster wound healing have made minimally invasive surgery (MIS) more popular. However, little research has compared the fixation results of minimally invasive chevron-akin (MICA) osteotomy thus far. In this study, the clinical and radiographic results of MICA with first metatarsal single- or dual-screw fixation are being examined.

**Methods:**

A total of 107 feet of 103 patients with mild to moderate symptomatic hallux valgus treated MICA from January 2018 to June 2020 were retrospective evaluated, with at least 12-months follow-up. 51 patients underwent single-screw fixation procedures and 52 patients received dual-screw fixation procedures. Patients were assessed preoperatively and at the final follow-up with radiographic measurements [hallux valgus angle (HVA), intermetatarsal angle (IMA) and distal metatarsal articular angle (DMAA)] and clinical scores (american orthopaedic foot and ankle society (AOFAS) forefoot score, visual analog scale (VAS) and Manchester-Oxford Foot Questionnaire (MOxFQ) scores). The coughlin satisfaction scores were also obtained.

**Results:**

Both groups showed significantly improved HVA, IMA and DMAA at the final follow-up (*P* < 0.001). Regarding clinical outcomes, the AOFAS, VAS and MOxFQ in two categories also significantly improved postoperatively (*P* < 0.001). There was no obvious difference in the clinical and radiographic outcomes between the two groups (HVA, *P* = 0.833; IMA, *P* = 0.073; DMAA, *P* = 0.35; AOFAS, *P* = 0.48; VAS, *P* = 0.86; MOxFQ, *P* = 0.87). However, the single-screw fixation group showed significantly lower operation time and less number of intraoperative fluoroscopy (*P* < 0.001). No serious complications were observed in either group. The single-screw fixation technique saves at least $1,086 compared with the dual-screw group.

**Conclusion:**

At the final follow-up, both the single- and dual-screw fixation groups had comparable good to excellent clinical and radiographic outcomes, as well as a similar incidence of complications. Additionally, the single-screw fixation group reduces overall surgical costs, number of intraoperative fluoroscopy and operational time.

## Introduction

Hallux valgus is a common forefoot deformity that usually presents as an exostosis of the first metatarsophalangeal joint and an inversion of the first metatarsal, with a prevalence of approximately 23%–35.7% (1), causing medial pain of the first metatarsophalangeal joint and even metastatic metatarsalgia. There are several surgical options available for hallux valgus treatment, but open surgery is still the predominant approach (2). In recent years, hallux valgus minimally invasive surgery (MIS) has been increasingly used in clinical practice due to its less invasive nature, faster recovery and comparable surgical results (3, 4). Hallux valgus MIS techniques are now in their third generation. The first and second generation techniques do not involve screws as internal fixation devices (5) and have a higher incidence of postoperative complications (6). The core of the third-generation hallux valgus MIS consists of a percutaneous osteotomy of the first metatarsal head and internal fixation with the fully threaded hollow screw.

The minimally chevron-akin (MICA) osteotomy is currently the most commonly used technique for third-generation (3G) hallux valgus MIS. Drs. Redfern and Vernois popularized and improved this procedure in Europe in 2008 (7). Compared to the open technique, the MICA has a lower incidence of postoperative stiffness and lessens postoperative discomfort (8, 9). Noteworthy, metalwork is most frequently used to hold and fix the osteotomy. Although these methods have been demonstrated to be successful, they do also have some drawbacks. In particular, there is a described incidence of subsequent implant removal due to irritation of surrounding tissues and migration of the implanted metalwork, which may need the use of additional equipment (10). The desired outcome is therefore to apply sufficient fixation with the least amount of implant.

The aims of this retrospective study were to compare the results of single- and dual-screw MICA for hallux valgus by evaluating the radiographic and clinical outcomes. Furthermore, a cost-effectiveness comparison between the two fixation types was also conducted.

## Methods

This was a retrospective comparative study carried from January 2018 to June 2020, 103 patients (107 feet) with mild to moderate hallux valgus that had been treated with MICA by the senior surgeon were included. The study was approved by the institutional review board of our institution, and written informed consent was obtained from all patients prior to the study.

Two case series were reviewed and compared in this study. One series was that of a surgeon who performs MICA using the first metatarsal single-screw fixation technique. The second series was that of using the first metatarsal dual-screw fixation technique. The inclusion criteria were as follows: (1) 18 < age < 65; (2) 15° < Hallux valgus angle (HVA) ≤ 40°; (3) 9° < Intermetatarsal angle (IMA) ≤ 16° (11); (4) Patients have been well compliant, accept the MICA technique and sign an informed consent form; (5) complete a minimum 2-year follow up; (6) No combined severe cardiopulmonary dysfunction, and able to tolerate surgery and anaesthesia. The exclusion criteria were as follows: (1) HVA > 40° or IMA > 16°; (2) Degenerative disease of the first metatarsophalangeal joint with severe restriction of joint movement; (3) Akin osteotomy only; (4) Patients with combined cardiovascular and cerebrovascular disease who cannot tolerate surgery; (5) Combined autoimmune disease or diabetes mellitus with poor glycaemic control; (6) Those with psychiatric disorders who are unable to cooperate.

### Operative technique and postoperative rehabilitation

The patient is placed in a supine position with three-quarters of the lower leg extended at the end of the bed and the foot resting on the mini C-arm (Mobile C-arm fluoroscopic x-ray system, SIEMENS). A combination of ultrasound-guided local nerve block combined with laryngeal mask anaesthesia is used and a tourniquet is tied and padded on the thigh.

**Figure 1:**
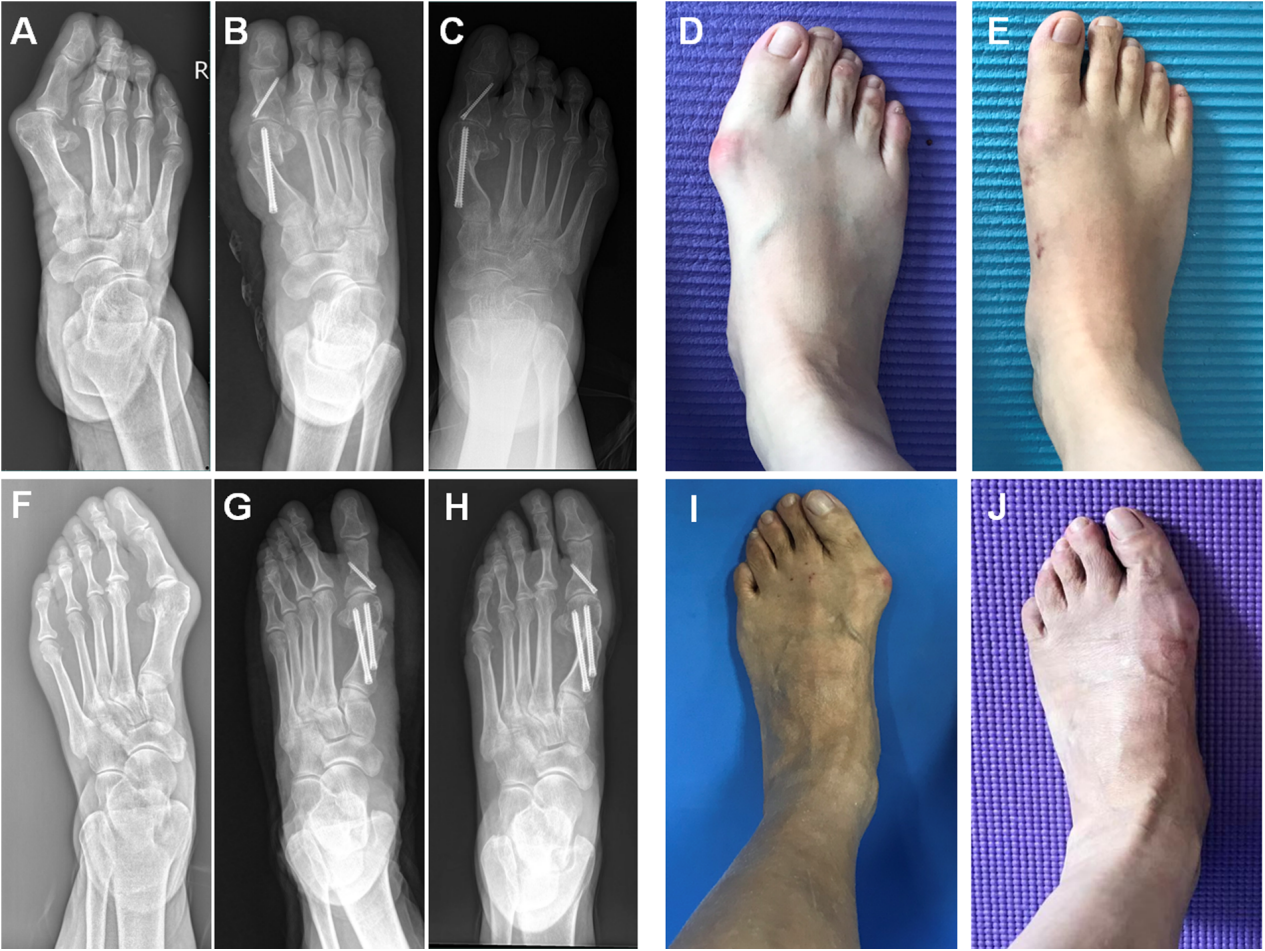
(**A,F**) Radiography showed moderate hallux valgus deformity. (**D,I**) Corresponding clinical photo. Radiography showed the correction of hallux valgus deformity after the single-screw fixation MICA (**B**) and the dual-screw fixation MICA (**G**). (**C,H**) 24 months after the operation and corresponding clinical photo (**E,J**).

A 5-mm incision was made at the first metatarsal head/neck junction, then a miniature periosteal stripper was used to separate the soft tissue. A low-speed, high-torsional minimally invasive power (Shanghai Bojin, 5,000 rmp) was selected to reduce thermal damage to the skin and soft tissues, and a 2*8 mm milling drill was used to perform a V-shaped osteotomy toward the second metatarsal head under image control. After the osteotomy was finished, the first metatarsal head was displaced laterally and assisted reset using a specially designed minimally invasive tool. A 5-mm incision was made at the slightly distal end of the first tarsometatarsal joint, a guidewire was inserted obliquely through this incision under image control. The wire penetrates the medial and lateral cortices of the proximal end of the osteotomy to reach the lateral cortex of the metatarsal head. Then a 4.0 mm fully threaded hollow screw (In2bones, French) is inserted under the guidance of the guidewire. In the dual-screw fixation group, the second screw is placed in the same manner and parallel to the distal end of the first screw Figure 1. The projection of the bone at the end of the osteotomy is cleaned with a grinding drill. A minimally invasive Akin osteotomy is performed on the first phalanx and a 3.0 mm screw is placed, if metatarsal osteotomy failed to completely correct the metatarsophalangeal joint match. Moreover, in patients with lateral tension and difficult repositioning of the first metatarsophalangeal joint, a 50 ml syringe needle was used to release the joint capsule and adductor hallucis on the lateral side of the first metatarsophalangeal joint prior to MICA. In patients with metastatic metatarsalgia, a minimally invasive distal metatarsal minimal osteotomy (DMMO) was performed with minimally invasive power without internal fixation. For patients with combined flexible flatfoot, minimally invasive subtalar joint arthroereisis is used. Minimally invasive gastrocnemius recession was performed in patients with partial gastrocnemius tendon tension.

A postoperative bandage is essential to maintain the position of the hallux and prevent chronic complications such as recurrence. A coil of bandage is placed between the flippers of the first and second toes, and another bandage is placed between the flippers of the 1st and 2nd toes through the ankle joint to hold the hallux in a neutral position for 6 weeks. Two weeks postoperative, the stitches are removed, and walking with walkers or forefoot decompression shoes is permitted. To avoid stiffness of the first metatarsophalangeal joint, full weight bearing is permitted if tolerated, and walking is encouraged. To progressively recover joint motion, light plantarflexion activities can be started. After 1 month, walking in sports shoes is permitted, followed by jogging after 3 months, and intense sports after four to 6 months. Patients are recommended to be reviewed and followed up at 6 weeks, 3 months, 6 months, 12 months and 24 months after surgery.

### Assessments

The visual analogue scale (VAS), the american orthopaedic foot and ankle society (AOFAS) forefoot scoring system and Manchester-Oxford Foot Questionnaire (MOxFQ) scores were used preoperatively and postoperatively respectively (5, 12), with the latter evaluating the clinical efficacy in terms of pain, function and strength lines, with a full score of a total score of 90–100 is considered excellent, 75–89 is considered good, 50–74 is fair and below 50 is considered poor. At the final follow-up, the mobility of the first metatarsophalangeal joint was assessed for joint stiffness. The Coughlin rating system was used to determine patient satisfaction levels (13). Patients who experienced few to no issues, little to no pain, ambulated without much difficulty, and were satisfied with the outcomes received a good to exceptional score. Radiographic assessment were as follows: HVA, IMA and DMAA. All radiographic parameters and clinical outcomes were measured by two orthopaedic surgeons.

### Statistical analysis

The Shapiro-Wilk method was applied to test for normality using SPSS 26.0 statistical software. Measures that conformed to a normal distribution were described as mean ± standard deviation (±s), and *t*-tests were performed between normally distributed data. The differences between the two groups of qualitative data such as sex and laterality were tested by chi-square test. Differences were indicated as statistically significant at *P* < 0.001.

## Results

The mean duration of follow-up was 20.98 ± 5.14 months in single-screw fixation group and 21.44 ± 4.10 months in dual-screw fixation group. The comparison of the baseline characteristics of the two groups was statistically insignificant (Table 1). The proportion of the release the joint capsule; distal metatarsal minimal osteotomy; minimally invasive subtatar joint arthrodesis and minimally invasive gastrocnemius recession in the two groups were 88.7% vs. 85.2%; 100% vs. 100%; 11.3% vs. 13.0% and 13.2% vs. 14.8%. At the end of follow-up, both groups had statistically significant improvements in the HVA, the first-second IMA and DMAA as compared to preoperative radiography (*P* < 0.001). The mean preoperative HVA, IMA and DMAA for the single-screw fixation MICA group were 33.98 ± 6.9°, 10.51 ± 3.0°and 13.99 ± 4.1°, respectively. At final follow-up, this improved to a mean HVA of 10.28 ± 5.11°, IMA of 4.72 ± 2.6° and DMAA of 4.79 ± 3.46° (*P* < 0.001). The mean preoperative HVA, IMA and DMAA for the dual-screw fixation MICA group were 34.15 ± 8.83°, 10.54 ± 3.0° and 16.16 ± 6.11°, respectively. At final follow-up, this improved to a mean HVA of 10.07 ± 5.18°, IMA of 3.9 ± 2.0° and DMAA of 5.73 ± 4.2° (*P* < 0.001). Both HVA, IMA and DMAA had comparable ultimate outcomes at the final follow-up when groups were compared (*P* = 0.833, *P* = 0.073 and *P* = 0.35, respectively). Table 2 lists the results of radiographic analysis. Figure 1 shows the postoperative radiographs of patients from each group at 2-year follow-up.

**Table 1:** Patient characteristics.

Characteristics	Single-screw	Dual-screw	*P* value
Age, mean ± SD, years	43.87 ± 17.80	44.58 ± 13.88	0.79
**Gender**			
Female	45	44	0.592
Male	6	8	
**Side of hallux vaglus**			
Right	28	25	0.499
Left	25	29	

**Table 2:** Comparison of patients based on the degree of preoperative deformity.

Characteristics	Single-screw	Dual-screw	*P* value
Preoperative HVA (°)	33.98 ± 6.9	34.15 ± 8.83	0.912
Postoperative HVA (°)	10.28 ± 5.11	10.07 ± 5.18	0.833
*P* value	<0.001	<0.001	
Difference	23.7 ± 7.5	24.08 ± 7.55	0.794
Preoperative IMA (°)	10.51 ± 3.0	10.54 ± 3.0	0.952
Postoperative IMA (°)	4.72 ± 2.6	3.9 ± 2.0	0.073
*P* value	<0.001	<0.001	
Difference	5.78 ± 3.33	6.64 ± 2.68	0.147
Preoperative DMAA (°)	13.99 ± 4.1	16.16 ± 6.11	0.12
Postoperative DMAA (°)	4.79 ± 3.46	5.73 ± 4.2	0.35
*P* value	<0.001	<0.001	
Difference	9.19 ± 4.7	10.42 ± 4.97	0.34
Preoperative AOFAS	56.49 ± 10.29	54.94 ± 11.74	0.53
Final AOFAS	90.25 ± 6.15	89.15 ± 8.58	0.48
*P* value	<0.001	<0.001	
Preoperative VAS	5.75 ± 1.63	5.20 ± 1.41	0.08
Final VAS	0.62 ± 0.56	0.65 ± 0.55	0.86
*P* value	<0.001	<0.001	
Preoperative MOxFQ	50.09 ± 13.28	51.94 ± 13.08	0.47
Final MOxFQ	12.85 ± 8.28	13.15 ± 11.03	0.87
*P* value	<0.001	<0.001	
Patient satisfaction, No.			0.175
Excellent	35	34	
Good	18	19	
Fair	0	1	
Poor	0	0	

HVA, hallux valgus angle; IMA, intermetatarsal angle; DMAA, distal metatarsal articular angle; VAS, visual analog scale.

Between the two groups, the preoperative ratings were comparable. Both groups had significantly better clinical results at the final follow-up (*P* < 0.001). AOFAS scores of two groups rising from 56.49 ± 10.29 to 90.25 ± 6.15 and 54.94 ± 11.74 to 89.15 ± 8.58, respectively. In the single-screw fixation group, the VAS and MOxFQ score decreased from 5.75 ± 1.63 to 0.62 ± 0.56 and 50.09 ± 13.28 to 12.85 ± 8.28; in the dual-screw fixation group, it decreased from 5.20 ± 1.41 to 0.65 ± 0.55 and 51.94 ± 13.08 to 13.15 ± 11.03. Clinical outcomes between the two groups were not substantially different (*P* > 0.05, Table 2). 53 feet in the single-screw fixation group reported excellent (66%) and good (34%) patient satisfaction, whereas 54 feet in the dual-screw fixation group reported excellent (62.9%), good (35.2%), and fair (1.9%) satisfaction.

In either group, there were no wound complications recorded. Stiffness of the first metatarsophalangeal joint was noted in 2 patients in single-screw fixation group (3.8%) and 3 in dual-screw fixation group (5.6%). Hallux valgus recurrence rates (5.7% and 3.7%) were comparable between groups receiving single or dual-screw fixation. Two patients in the single-screw fixation group (3.8%) and 3 patients in the dual-screw fixation group (5.6%) also reported numbness. One additional patient had persistent dysesthesias over the dorsomedial foot in dual-screw fixation group. One foot in the dual-screw fixation group had skin irritation which improved after screw removal. In neither group were any cases of avascular necrosis, nonunion, or dorsal malunion of the distal fragment described.

A cost analysis was also performed comparing the material costs of two different types of surgical fixation. The average cost of a screw was $1,085 and the single-screw fixation technique was found to be more cost-effective. the single-screw fixation group required an average of 15.42 ± 3.00 numer of intraoperative fluoroscopy and took 46.87 ± 5.23 min on average to complete, both of which were significantly less than the average for the dual-screw fixation group (Table 3, *P* < 0.001).

**Table 3:** Perioperative outcomes.

Characteristic	Single-screw	Dual-screw	*P* value
Operative time (min)	46.87 ± 5.23	56.59 ± 5.20	<0.001
Number of intraoperative fluoroscopy	15.42 ± 3.00	18.72 ± 2.06	<0.001

## Discussion

Being the first comparison results of single/dual-screw fixation for MICA technique in our region, our study offers a novel viewpoint on third-generation MICA procedures in the Asian population. A minimum follow-up period of 2 years was successfully attained, allowing examination of short- to medium-term outcomes and complications. All surgeries were performed by same two surgeons using the same method in an effort to minimize the impact of intersurgeon technique variability on our outcomes. Standardization was also applied to postoperative treatment, procedure, and follow-up.

The demand for MIS has recently surged. There are some suggested advantages, such as a quicker healing time and a lower chance of infection. We do believe that the high union rate and stability of the MICA osteotomy are largely attributable to the bicortical structure of the proximal metatarsal screw, which was first mentioned by Redfern et al. (7), and the minimal soft tissue disturbance caused by the percutaneous operation. However, there are few to no articles on the subject of comparative outcomes of single/dual-screw fixation for MICA.

At the final follow-up in the current study, there were no differences in postoperative HVA, IMA or DMAA between the two groups. It has been established that patient satisfaction following hallux valgus surgery is inversely correlated with angular correction. Recurrence is linked to inadequate DMAA correction, especially in adolescents with hallux valgus (14). The average IMA correction served as a gauge for the potential of metatarsal osteotomy correction. The sesamoids should be completely repositioned as a consequence of a full decrease of the IMA. A correlation between the IMA and sesamoid location has been established in recent research in both hallux deformity and after hallux valgus treatment (15). In our investigation, we discovered that IMA corrections in both groups were good (Table 2). Analysis of the HVA also revealed a similar correction with barely any loss of correction in both groups, similar to the IMA. The HVA is affected directly by a phalangeal osteotomy and indirectly by the quality of the lateral release and the metatarsal osteotomy. The HVA correction in our study (Table 2) is in good agreement with the results that have been reported in the literature (16). Additionally, these findings suggest that one screw fixation would be adequate to ensure the stability of the osteotomy site, and the clinical outcomes dramatically improved. It is commonly recognized that clinical scores significantly improve following hallux valgus surgery (17). Using the MICA approach, the midterm findings of Hernandez et al. had promising clinical results. At 59.1 months following surgery, the AOFAS score went from 62.5 to 97.1 (18). Additionally, this discovery was also revealed during retrospective analyses of MIS hallux valgus operations. Our statistical findings support this assertion even more (Table 2). The British Orthopaedic Foot & Ankle Society has approved MOxFQ as a way to assess surgical results in foot and ankle surgeries. Significant improvement was seen in both groups when VAS and MOxFQ was evaluated (Table 2), which may be related to the slight irritation of soft tissue. Overall, similar to the earlier study (19), there was no appreciable difference between the two groups in clinical and radiological outcomes.

Recurrence rates following surgery for hallux valgus have been estimated to reach 16% (20). Recurrent hallux deformity can be very difficult to treat since there is a lot of scar tissue left over and there are more problems than with primary surgery (21). This rate may be further decreased by the current MICA technique, which has a better screw fixation in the bone, although it has not yet been well researched in revision surgery. The recurrence rate in our study was essentially the same for both groups. The preservation of soft tissue is the fundamental technical benefit of MIS. In MICA, insufficient joint and work area cleaning may leave behind bone fragments that trigger an inflammatory reaction, leading to discomfort, fibrosis, and stiffness (22). In this study, compared to the single-screw fixation group, the dual-screw fixation group entailed greater soft tissue and bone manipulation and had a higher incidence of joint stiffness. The most frequent complication following MICA in the earlier trials was screw irritation. Our 1.9% screw removal rate in the dual-screw fixation group wasn't exceptionally high compared to earlier MICA experiments. Holme et al. reported a 10% complication rate, comprised of 4 patients who needed to have hardware removed because it had inflamed their soft tissues. It was proposed that the usage of oblique headless screws was the cause of their hardware removal rate being lower than previously observed (24%). Screw irritation was not found within the single-screw fixation group. Nerve discomfort is a rare consequence following this surgery because of the percutaneous aspect of the technique, which raises concerns about possible damage to the dorsal medial cutaneous nerve. Jowett and Bedi (23) did note two patients who exhibited scar sensitivity (24, 25). Less than 3% of nerve injuries were reported by Redfern et al. (7). In this study, both of our groups experienced the same rate of postoperative nerve paresthesias. Problems with wound healing and infections are also infrequently discussed in the MICA literature. In their research of 120 patients, Jowett and Bedi (23) identified 2 incidences of wound infections. Such complications did not originate in either group in our series.

The first metatarsal single-screw fixation group strategy had the lowest proportion of implants, which was a measure of the overall cost of the surgery, compared to dual- screw fication group, it is even more cost-effective than other approaches (26). MIS is reported to be faster than open scarf-Akin (27, 28). This study has demonstrated that clinical and radiological results are comparable with two groups. However, Operation time and number of intraoperative fluoroscopy were considerably reduced in the single-screw fixation group compared to the dual-screw fixation group (Table 3, *P* < 0.001).

This study has several limitations. To start, since the study only included mild to moderately patients, it is not impossible to draw conclusions about the effectiveness of treating severely patients. Second, our study population involves a small number of subjects. This was caused by a number of factors, e.g., patients who were excluded for missing follow-up appointments or having follow-up periods that were shorter than 2-years were deemed to have incomplete data sets. Third, our overall patient follow-up time is another drawback because this study only looked at short- to medium-term patient outcomes. As a result, we are unable to make any judgments on the long-term effects of this approach. At last, fully threaded hollow screws were used in this study. It is not clear whether other screws, such as double-headed compression screws, can also achieve stability. These restrictions should be considered in future study, and mitigating strategies should ideally be included.

## Conclusion

In addition to offering outcomes that are equivalent to those of dual-screw MICA for the first metatarsal, the single-screw technique also offers the benefit of lowering overall surgical costs, fluoroscopy number during surgery and operational time. We recommend single-screw MICA as a viable treatment option.

## Data Availability

The raw data supporting the conclusions of this article will be made available by the authors, without undue reservation.
